# Enhancing COVID-19 CT Image Segmentation: A Comparative Study of Attention and Recurrence in UNet Models

**DOI:** 10.3390/jimaging9120283

**Published:** 2023-12-18

**Authors:** Rossana Buongiorno, Giulio Del Corso, Danila Germanese, Leonardo Colligiani, Lorenzo Python, Chiara Romei, Sara Colantonio

**Affiliations:** 1 Institute of Information Science and Technologies, National Research Council of Italy (ISTI-CNR), 56124 Pisa, PI, Italy; giulio.delcorso@isti.cnr.it (G.D.C.); sara.colantonio@isti.cnr.it (S.C.); 2 Department of Translational Research, Academic Radiology, University of Pisa, 56124 Pisa, PI, Italy; leonardo.colligiani@studenti.unipi.it; 3 2nd Radiology Unit, Pisa University Hospital, 56124 Pisa, PI, Italy; lorenzo.python@stud.unifi.it (L.P.);

**Keywords:** COVID-19, segmentation, deep learning, convolutional neural networks, UNet, attention mechanism, recurrency

## Abstract

Imaging plays a key role in the clinical management of Coronavirus disease 2019 (COVID-19) as the imaging findings reflect the pathological process in the lungs. The visual analysis of High-Resolution Computed Tomography of the chest allows for the differentiation of parenchymal abnormalities of COVID-19, which are crucial to be detected and quantified in order to obtain an accurate disease stratification and prognosis. However, visual assessment and quantification represent a time-consuming task for radiologists. In this regard, tools for semi-automatic segmentation, such as those based on Convolutional Neural Networks, can facilitate the detection of pathological lesions by delineating their contour. In this work, we compared four state-of-the-art Convolutional Neural Networks based on the encoder–decoder paradigm for the binary segmentation of COVID-19 infections after training and testing them on 90 HRCT volumetric scans of patients diagnosed with COVID-19 collected from the database of the Pisa University Hospital. More precisely, we started from a basic model, the well-known UNet, then we added an attention mechanism to obtain an Attention-UNet, and finally we employed a recurrence paradigm to create a Recurrent–Residual UNet (R2-UNet). In the latter case, we also added attention gates to the decoding path of an R2-UNet, thus designing an R2-Attention UNet so as to make the feature representation and accumulation more effective. We compared them to gain understanding of both the cognitive mechanism that can lead a neural model to the best performance for this task and the good compromise between the amount of data, time, and computational resources required. We set up a five-fold cross-validation and assessed the strengths and limitations of these models by evaluating the performances in terms of Dice score, Precision, and Recall defined both on 2D images and on the entire 3D volume. From the results of the analysis, it can be concluded that Attention-UNet outperforms the other models by achieving the best performance of 81.93%, in terms of 2D Dice score, on the test set. Additionally, we conducted statistical analysis to assess the performance differences among the models. Our findings suggest that integrating the recurrence mechanism within the UNet architecture leads to a decline in the model’s effectiveness for our particular application.

## 1. Introduction

Coronavirus disease 2019 (COVID-19), caused by the Severe Acute Respiratory Syndrome Coronavirus 2 (SARS-CoV-2), has led to a global health crisis of pandemic proportions. The SARS-CoV-2 infection can result in mild symptoms affecting the upper respiratory tract, similar to other viral respiratory diseases, but it can also rapidly lead to severe pneumonia [1]. Consequently, its quick progression highlights the critical significance of early diagnosis.

Conventional Reverse Transcription-Polymerase Chain Reaction (RT-PCR) was the only way to detect the disease in the early months of the pandemic, but it may produce false negative results (low sensitivity), especially in the early stages of infection, when the viral load is too low and insufficient cellular material may be present in the sample for effective virus detection [2].

**Figure 1:**
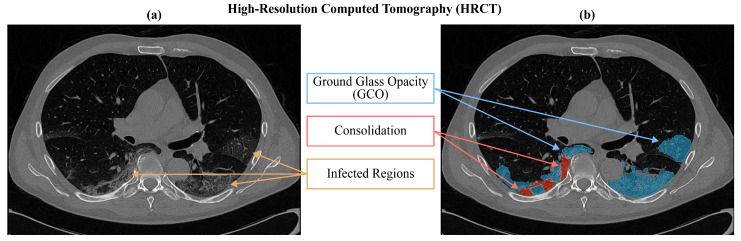
Manifestations of COVID-19-infected regions on an HRCT of a confirmed patient. Panel (**a**) shows the original grey-level intensities, while in Panel (**b**) the infected regions are manually enhanced by radiologists (GGO in blue and Consolidation in red).

To overcome the major limitations of RT-PCR, High-Resolution Computed Tomography (HRCT) of the chest has been adopted as an alternative technique to visually detect viral infections, especially in compromised, hospitalized patients ([3,4]). In Figure 1, two distinct diseased patterns are indicated: an area of increased attenuation and hazy density on the lung lobes, known as Ground Glass Opacity (GGO), and many bilateral areas of Consolidation, which are portions of typically compressible lung tissue that are filled with fluid instead of air [5,6]. The accurate detection of these two distinct abnormal features is the main goal of HRCT, since detecting and quantifying such findings in terms of lung involvement is a key step in identifying significant information for the classification of the disease even in patients with a negative RT-PCR test [7]. However, as can be seen in Panel (a) of Figure 1, the patterns are scattered with undefined contours and often present little contrast to the surrounding healthy tissue.

Indeed, the segmentation of HRCT images, which means the manual delineation and quantification of the pathological lung regions from the imaging data, was revealed to be a challenging and time-consuming task, not only for this reason, but also due to the high number of cases to report, the magnitude of the imaging data, and the similarity of COVID-19 patterns with other types of pneumonia [8]. A modern solution to this challenge is the integration of automated segmentation using Artificial Intelligence (AI), specifically methods based on Deep Learning (DL) [4,9] and Convolutional Neural Networks (CNNs) [10,11,12].

Although there are ad hoc models developed for COVID-19, such as Inf-Net and Semi-Inf-Net by Fan et al. [13], nCovSegNet of Liu et al. [14], and LungINFseg in [15], the best results for segmentation of these pathological zones were obtained from UNet variants. In fact, many researchers have developed UNet-based models to detect COVID-19-related infections with very promising results (for example, in [16,17]). In [18], a UNet-based framework for COVID-19 segmentation exploiting a novel connectivity promoting regularization loss function was proposed. Even in the MICCAI-endorsed challenge on COVID-19 segmentation, all top 10 models were UNet-based: among all, the best performing model was a high-resolution UNet with extensive data augmentation and instance normalization ([19]).

Many variants of UNet have emerged from its success, including the UNet with attention (Attention-UNet) [20], the Recurrent Residual convolutional UNet (R2-UNet) [21], and the Recurrent and Residual convolutional UNet with attention (R2-Attention UNet) [22].

Generally speaking, the attention mechanism enables a DL model to selectively focus on relevant regions, improving its ability to identify and segment structures of interest in complex and varied images. The attention modules embedded in CNNs generate attention maps that highlight the importance of different spatial locations in the feature maps, improving the overall sensitivity to subtle and dispersed features and enhancing the capability to handle variations in the size, shape, and appearance of structures.

Regarding medical image segmentation, one of the earliest applications of the attention mechanism in CNNs was for segmentation of the pancreas in CT images, but still, new UNet-based architectures incorporating attention modules are used for segmentation of MRI images, e.g., for segmentation of a brain tumor [23] or an aneurysm [24], and CT images, e.g., for liver [25] and lung detection.

As far as the segmentation of COVID-19 HRCT data is concerned, various examples of attention mechanisms embedded in UNet models can be found in the literature as well. In [26], the authors used an attention mechanism by introducing attention gates in the network, designing Attention Gate-Dense Network-Improved Dilation Convolution-UNet (ADID-UNet). In [27], spatial and channel attention modules were incorporated. Furthermore, other solutions aimed at reducing the false positive rate used the attention mechanism, for example, by applying a dilated dual attention mechanism (D2A-UNet) [28] or a combination of attention with a boundary loss function to deal with small and unbalanced data [29]. Other novel models born to segment COVID-19 infections with a UNet architecture and attention mechanism were proposed in [30,31]. The former used a UNet-like pyramid encoder and an Attention-UNet-like double decoder to design PDAtt-UNet to segment COVID-19 infections and lungs, while the latter consisted of a modified UNet that combines the squeeze-and-attention and dense atrous spatial pyramid pooling modules to fuse global context and multi-scale information.

Recurrent and residual mechanisms are two important architectural components that have been integrated into CNNs for medical image segmentation.

The recurrent mechanism is designed to capture sequential patterns in data. In fact, while traditional CNNs are primarily designed for grid-like data, recurrent mechanisms allow for the network to maintain and update a hidden state that can carry information across different parts of the input sequence. This can be useful for capturing long-range dependencies in images with structures and patterns that span large spatial areas, enabling the model to consider contextual information across the entire image. Moreover, recurrent connections can be used to iteratively refine predictions, especially when dealing with complex structures or fine details.

The residual mechanism introduces shortcut connections that bypass one or more layers in the network to address the vanishing gradient problem. In addition, residual connections allow for the network to reuse features from earlier layers, aiding in the learning of hierarchical representations. This is valuable in medical images where different levels of abstraction may be necessary for obtaining accurate segmentation.

When dealing with medical image segmentation, combining recurrent and residual mechanisms can be a powerful strategy, leveraging both sequential dependencies and the ability to train deep networks effectively. These hybrid architectures aim to capture both spatial and sequential information, improving the model’s ability to handle the complexities of medical images.

Even though early experiments were performed with recurrence and residual modules embedded in an UNet-based model for some medical image tasks, such as blood vessel segmentation of retina, skin, and lung segmentation [21], the potential of R2 networks in COVID-19 pattern detection has not been extensively investigated in the literature, and only a few studies have shown promising but preliminary results, both in segmentation [32] and classification (ProgNET) [33].

Similarly, the combination of recurrence, residual and attention mechanisms (R2-Attention UNet) applied to COVID-19 segmentation remains an almost unexplored topic; in fact, only residual networks with attention (thus without recurrence) have been successfully applied to this challenging topic (for example, Residual Attention U-Net [34] and CARes-UNet [35]).

Given the potential of R2 and attention networks, the lack of peer-reviewed comparative articles in the literature is a major limitation in the selection of the most promising model for future studies. Some works have been concerned with providing a review of the existing architectures mentioned above and their application, such as in [36] and in [37]. Several DL models on COVID-19 image segmentation were compared in [38], but none included either attention or recurrence and residual mechanisms. In fact, to the best of our knowledge, no one has developed a rigorous comparison with a k-fold cross-validation scheme, a pre-processing workflow, and an evaluation process with several exhaustive 2D and 3D metrics between these three computational mechanisms and the simple UNet that can be associated with human cognitive mechanisms aimed at understanding complex images.

In addition, as shown in Table 1, most analyses use public databases that are limited in size, both in terms of patients and number of labeled images (e.g., [27,28,32]). Moreover, some works on COVID-19 segmentation have no cross-validation (e.g., [17,18,19,20,21,22,23,24,25,26,27,28,33]), and result in high values of the Dice score if evaluated on small datasets.

In this work, we compared the performances of UNet, R2-UNet, Attention-UNet, and R2-Attention UNet on the binary segmentation of COVID-19 infections. This comparison was carried out using a novel dataset of 90 HRCT volumetric scans and (corresponding to 90 patients and 26,683 images) of patients diagnosed with COVID-19. The dataset was collected within the regional project “OPTIMIZED —An Optimized Path for the Data Flow and Clinical Management of COVID-19 Patients”, funded by the Tuscany region. The project, started in 2021 and still ongoing, aims to create an optimized pathway for the data flow and clinical management of COVID-19 patients, based on imaging, hematological and clinical data. On the HRCT imaging data collected within the project, we trained and tested the mentioned UNet variants under equal computational resources by setting up a five-fold cross-validation and assessing the strengths and limitations of these models in terms of the Dice score, Precision, and Recall considering both the single 2D images extracted from the volumes and the whole 3D volumes of each patient.

The paper is organized as follows: in Section 2, we present the dataset we used in this work and the methodology we followed to customize, train, test, and compare the different architectures; then, in Section 3, the experimental setup and results are described. Finally, Section 4 discusses and concludes the paper.

## 2. Data and Methods

**Figure 2:**
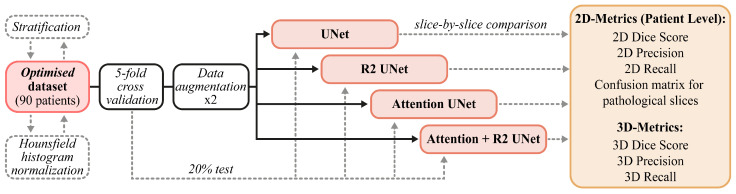
Schematic summary of the pipeline we followed: We started by describing the internal and external datasets, then moved on to the description of the models before showing the training and test set-up.

The data and methods described in this section are briefly summarized in Figure 2. In Section 2.1, we report on the provenance and characteristics of the internal and external data used in our experimental activities. Next, in Section 2.4, we describe the customized models that we developed, paying particular attention to the main components of each architecture. Finally, in Section 2.5 and Section 2.6, we describe both the training and cross-validation schemes and the metrics chosen for the evaluation of performances (Section 2.6).

### 2.1. Data

The following subsections elucidate the dataset employed in this study, utilized for training, validation, and testing of the models.

#### “OPTIMIZED” Dataset

Between February 2021 and April 2022, the Optimized project gathered 90 HRCT volumetric scans of 90 patients diagnosed with COVID-19 at the Pisa University Hospital, including 22 retrospective cases (hospitalized between June and September 2020) and 68 prospective cases (hospitalized between February 2021 and April 2022).

The Ethical committee verified the study’s compliance with the Standards of Good Clinical Practice of the European Union and with the ethical principles expressed in the Declaration of Helsinki, as stated in the consent for publication signed by each patient enrolled in the project (approval code 19275, approval date: 25 February 2021). After being collected, each HRCT scan underwent an anonymization process in order to remove all the personal information associated with the patient.

The dataset consists of a total of 26,683 2D slices extracted from all volumes, each with a resolution of 512 × 512 pixels per image.

In Table 2, the main data characteristics, expressed as median values and interquartile ranges (IQR), are reported.

As for ground truths, we obtained segmentation masks as an agreement among three expert radiologists, two of whom had five years of experience and one had more than ten years of experience. The process consisted essentially of three main steps. First of all, preliminary segmentation masks were provided by one of the two youngest radiologists. He used UIP-net [39], which is an encoder–decoder convolutional neural network trained from scratch for the segmentation of typical radiological patterns of Idiopathic Pulmonary Fibrosis (IPF). Since both IPF and COVID-19 manifest with interstitial lung abnormalities in chest CTs, the radiologist used segmentation provided by UIP-net as a preliminary mask to facilitate his manual work. Once the masks from UIP-net were obtained, he proceeded with the second step by manually refining the results and adding consolidations. The resulting segmentation masks were then checked by the second youngest radiologist who confirmed and corrected in case of error the segmentation provided by his colleague. To carry out this operation, they both used 3D Slicer (https://www.slicer.org (accessed on 15 December 2023) ), an open-source software for visualization, segmentation, registration, and analysis of biomedical images. The third phase consisted of the final review of the masks provided by the two radiologists by the senior radiologist, which produced our final ground truth consisting of a single binary mask containing both GGO and consolidation masks shown in Figure 1.

### 2.2. Data Normalization and Augmentation

**Figure 3:**
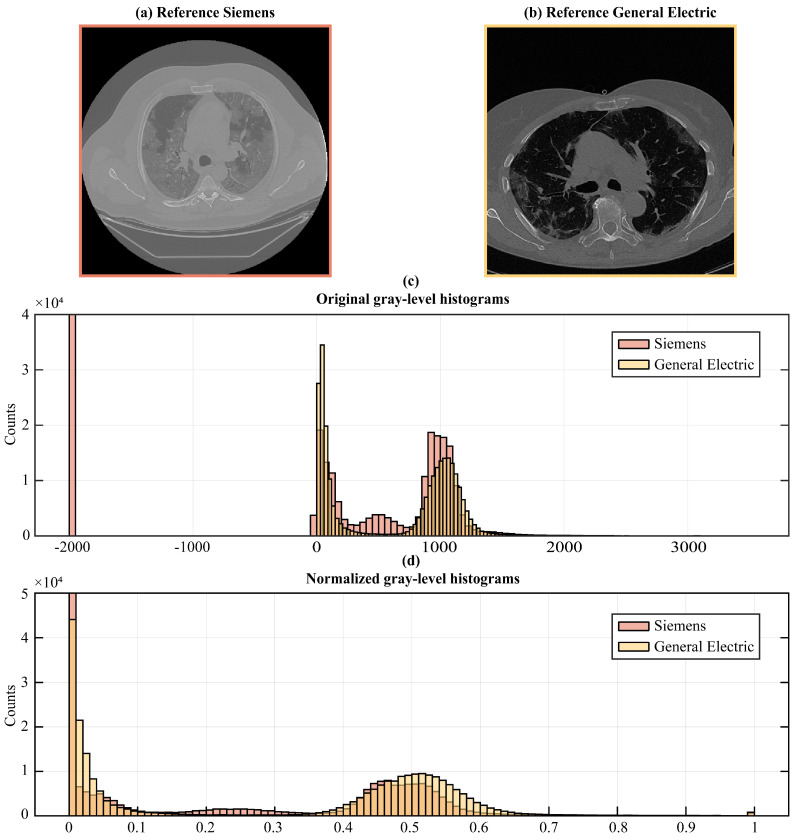
An example of (**a**) an image from the internal and (**b**) the external dataset. The two images are distinguished by gray-level distributions (**c**); thus, a preliminary step consisting of a histogram matching operation was necessary (**d**).

To remedy the intrinsic variability in image acquisition and intensity values among different scanners and settings, we followed image normalization procedures, thus enabling the direct comparison of image data retrieved from diverse sources (see Figure 3).

First, we transformed the DICOM pixel values into Hounsfield units (HU) to account for the physical properties of the tissues and establish a common scale across all the images, since HU is a scale used in CT imaging to quantify radiodensity. To transform DICOM pixel values to HU, we accessed the DICOM pixel values (PV) from the image data and extracted the DICOM Rescale Slope ($R_{sl}$) and Rescale Intercept ($R_{int}$) included in the metadata information. These parameters are necessary for the linear transformation from DICOM pixel values to HU we performed using the following formula:

(1) $$HU=PV\times R_{sl}+R_{int}.$$

This linear transformation scales the pixel values to Hounsfield Units based on DICOM metadata.

Once transformed, we applied clipping to restrict the HU values within a specific range, [−1000HU; +1000HU], encompassing the gray-level spectrum from lung signal to bone density, thus eliminating values outside the range of our interest, to guide the network attention to the correct gray-scale range. This means that we set all pixels that had HU values greater than +1000 to +1000 and all pixels lesser than −1000 to −1000. This step was essential for ensuring the stability of the models, particularly when confronted with variations in image intensity scaling.

Finally, we rescaled the obtained HU values to fit within the normalized range of [0, 1] in order to speed up model convergence and to guarantee its stability during training. The rescaling operation was performed according to the following Equation (2):

(2) $$I_{rescaled}=l_{b}+\frac{\left(I-I_{min}\right)}{I_{max}-I_{min}}\times \left(u_{b}-l_{b}\right).$$

In Equation (2), $l_{b}$ and $u_{b}$ represent, respectively, the lower and upper bounds of the range of values in which the pixels are to be rescaled (in this case $l_{b}=0$ and $u_{b}=1$), while $I_{min}$ and $I_{max}$ are the current minimum and maximum values of pixels in the image.

Regarding data augmentation, we used a horizontal flip on the fly, which is a state-of-the-art technique that allows us application of data augmentation in real time during training of the model. This reduces the need for storage, allows for dynamic augmentation where the augmentation parameters are randomized for each batch, adding more variability to the training data, and, finally, increases efficiency because on-the-fly data augmentation can be more computationally efficient, particularly when working with large datasets.

We applied the horizontal flip in order to perform a rigid transformation compatible with the view of our data, which was the axial one. To achieve that, we reversed the order of the columns of each image, thus producing a mirrored version of the original image along the vertical axis (i.e., from right to left). In this way, we doubled the number of samples provided to each model during the training since we applied data augmentation only on the images belonging to the training set, therefore keeping those of validation and test sets unchanged.

### 2.3. UNet Architecture

**Figure 4:**
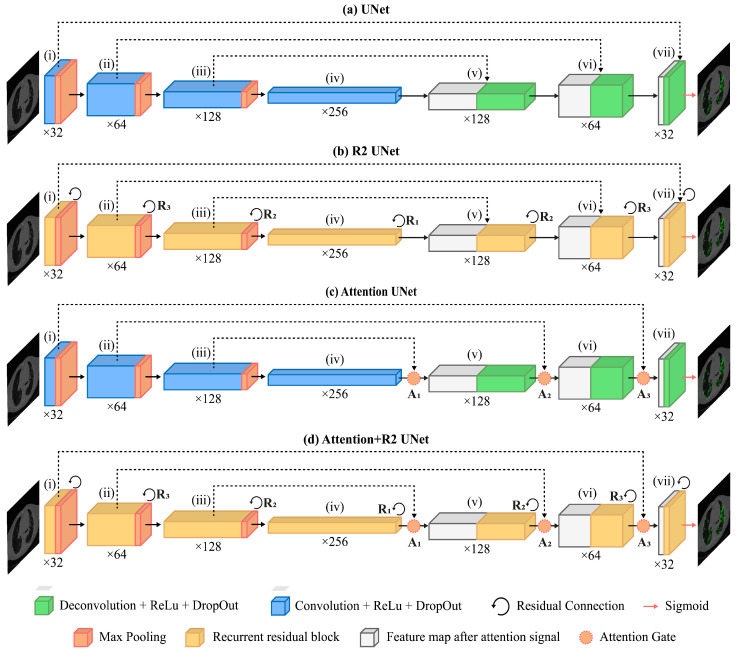
Schematics of the UNet-based models trained and tested in this work: UNet (**a**), R2-UNet (**b**), Attention-UNet (**c**), and R2-Attention UNet (**d**).

UNet is a Fully Convolutional Network (FCN) designed for medical image segmentation composed of an encoder and a decoder that offer it its distinctive U-shaped structure (Figure 4a).

For this study, the architecture was customized. The input layer took as input a gray-scale 2D image with 512 × 512 pixels. The encoding path involved repeated application of two 3 × 3 convolutions, each followed by a Rectified Linear Unit (ReLU) activation and a max pooling operation. This process doubled the number of feature maps for each convolutional layer along the down-sampling path, ranging from 32 to 256 maps.

Along the decoding path, starting from 256 maps, each 2 × 2 convolutional layer halved the number of feature maps. At each stage of the decoding path, skip connections were employed to pass the features from the encoder to the corresponding decoder path through concatenation. This allowed the recovery of spatial information lost during down-sampling operations. After concatenation, the resulting feature maps underwent two consecutive 3 × 3 convolutions, each followed by a Rectified Linear Unit (ReLU) activation.

The sigmoid activation function was used for the last convolutional layer, consisting of a 1 × 1 convolution used to map each feature vector to the desired number of classes, thus returning a 512 × 512 map as the binary mask discriminating the diseased tissue from the healthy one.

In Table S1, in Supplementary Materials, we reported the transformation functions layer by layer of the UNet architecture.

### 2.4. UNet-Derived Models

An overview of architecture objects of comparison used in this work, which are sourced from UNet, is given in the next subsections. We describe the Recurrent Residual Convolutional Neural Network based on UNet (R2-UNet) in Section 2.4.1, the UNet with the addition of attention gates (Attention-UNet) in Section 2.4.2, and finally the Recurrent Residual Convolutional Neural Network based on UNet with the addition of attention gates (R2-Attention UNet) in Section 2.4.3. In addition, the main components of the several models are described in order to highlight their contribution to the standard UNet architecture.

#### 2.4.1. R2-UNet Architecture

We obtained R2-UNet by adding recurrent residual convolutional blocks, explained in the following paragraph, at each stage of the architecture of UNet (Figure 4b). We set R2-UNet so that it takes as input data single-channel, gray-scale, 2D images with 512×512 pixels. The depth of the encoder and the decoder path was set equal to 3.

As mentioned above, every stage of the encoding and decoding paths consists of a recurrent residual convolutional block with three recurrent convolutional layers, each performing a convolution followed by ReLU activation function. To increase the ability of the model to integrate contextual information, residual connections were added to each recurrent convolutional layer, with a number of discrete time steps equal to 2, in order to recursively process the input only once at each stage. The recurrent residual convolutional blocks and their functioning are described in detail in the following paragraph.

In Table S2, in Supplementary Materials, we reported the transformation functions layer by layer of R2-UNet architecture.

Each recurrent residual convolutional block consists of two recurrent convolutional layers that evolve over two discrete time steps *T*. This means that each recurrent convolutional layer performs *T* convolutions followed by ReLU activation function. We set *T* equal to 2; thus, at time step t=0, only the input of the block is convoluted; for t=1, the convolution is with concatenation, which represents residual connection of the block input and the result of the previous step; see Equation (4). Finally, the output of the entire block consists of the concatenation between the input of the recurrent residual block and the output of the last recurrent convolutional layer (i.e., at time step t=1) (see Equation (3)).

Formally, considering the $u_{l}$ input sample in layer *l* of the recurrent residual block and a pixel located at (i,j) in an input sample on the $k_{t}h$ feature map in the recurrent convolutional layer, output $z_{ijk}$ at time step *t*, if t>0, of the recurrent convolutional layer can be expressed as follows:

(3) $$z_{ijk}\left(t\right)=w_{k}^{f}\times u_{l}^{\left(i,j\right)}\left(t\right)+w_{k}^{r}\times u_{l}^{\left(i,j\right)}\left(t-1\right)+b_{k}.$$

In the equation, $u^{\left(i,j\right)}$(*t*) and $u^{\left(i,j\right)}$(t−1) denote the feed-forward and recurrent input, respectively, which are the vectorized patches centered at (i,j) of the feature maps in the current and previous layers, respectively. $w_{k}^{f}$ and $w_{k}^{r}$ denote the feed-forward and recurrent weights, respectively, and $b_{k}$ is the bias. The output of the last recurrent convolutional layer $z_{ijk}\left(t\right)$ is activated by a ReLU function, $f(z_{ijk}\left(t\right))$, and the output $u_{l+1}$ of the entire recurrent residual convolutional block, given the input of the block $u_{l}$, can be expressed as follows:

(4) $$u_{l+1}=u_{l}+f\left(z_{ijk}\left(t\right)\right).$$

#### 2.4.2. Attention-UNet Architecture

We included Attention Gates in the decoding path of the UNet architecture (as described in more detail below) in order to identify the salient image regions and amplify their influence while suppressing the irrelevant and confusing information. This was performed to enforce a more focused use of feature maps. As in the previous cases, Attention UNet takes as input data single-channel, gray-scale, 2D images with 512 × 512 pixels, and the depth of both the encoder and the decoder paths was set equal to 3.

In Table S3, in Supplementary Materials, we reported the transformation functions layer by layer of Attention-UNet architecture.

An AG is put on each skip connection that passes the feature maps from a downsampling layer to the corresponding upsampling one (see Figure 4c). It is used to prune irrelevant and noisy activations in the stack of feature maps (i.e., the light blue one) that are concatenated with the feature maps obtained by upsampling those of the previous layer (i.e., the light gray one).

For example, considering layer (v), the corresponding AG takes in input the features maps of the previous layer (iv) and those from the corresponding downsampling one (iii). These stacks of features are first convoluted with a 1 × 1 kernel to shrink all the maps into a stack with a fixed size *Nf* with *Nf* computed as a quarter of the number of feature maps of (iv) so as to match, after the concatenation, the number of feature maps of (iv). Then, they are concatenated and passed through a ReLU activation layer and convoluted again with kernel 1 × 1 × 1 to obtain a single mask containing attention coefficients for each pixel. Attention coefficients tend to have significant values in target regions and small values in background ones, so as to improve the accuracy of segmentation. After that, they are passed through a sigmoid activation layer. The resulting mask is used to multiply element-wise the feature maps from (iii).

#### 2.4.3. R2-Attention UNet Architecture

Inspired by the work of Zuo and colleagues [22], to obtain R2-Attention UNet, we modified the architecture of UNet by inserting recurrent residual convolutional block (see Figure 4d) at each stage of the architecture, and AGs on the stages of the decoding path. As for R2-UNet and Attention-UNet, R2-Attention UNet took as input data single-channel, gray-scale, 2D images with 512×512 pixels, and the depth of the encoder and the decoder path was equal to 3.

Every stage of the encoding and decoding paths consists of a recurrent residual convolutional block with three recurrent convolutional layers, each performing a convolution followed by ReLU activation function. Recurrent connections were added to each recurrent convolutional layer.

In Table S4, in Supplementary Materials, we reported the transformation functions layer by layer of R2-Attention UNet architecture.

### 2.5. Training and Cross-Validation Scheme

For all the models, we selected the binary cross-entropy as a loss function, and the Adam Optimizer was used as the optimization algorithm with a learning rate equal to 0.001, the exponential decay rates for the moving average of the gradient equal to 0.9 and the squared gradient equal to 0.999. The batch size was set to 5.

The training run on Keras (version 2.3.1) and TensorFlow frameworks (version 1.14.0) was coded in Python 3.6. All experiments were performed under Windows 10 OS on a machine with CPU Intel(R) Core(TM) i7-10700F CPU @ 2.90 GHz, GPU NVIDIA GeForce GTX 1650, and 32 GB of RAM.

We set the number of epochs to 80 and saved the trained models at each epoch to test their performance afterward. Specifically, we implemented early stopping by calculating the validation loss after each epoch and defining a patience (i.e., the number of epochs to wait before stopping training if no improvement in performance is found on the validation set) equal to 20. Then, after saving the weights at each epoch, for each model, we ran the test by loading the saved weights at the epoch when the model reached the lowest loss on the validation set out of 20 epochs.

In order to make the training independent from the data split, we performed a k-fold cross-validation on the dataset described in Section 2.1. There were 90 patients in total, 18 patients in each fold. We chose k=5, and we set up the k-fold cross-validation so that each patient was either in the validation or the training set. In addition, because the average volume of the diseased regions can vary greatly between cases, we stratified them based on the average area of the diseased regions, expressed in mm^3^. In this way, the stratification ensures that for each fold there is a proportional number of cases with diseased regions of different sizes. We trained each model on 3 folds, validated it on 1 fold, and then tested it on the leftover fold.

### 2.6. Evaluation Metrics

To analyze the performance of the networks, we decided to use metrics defined on slices (2D) as well as on the entire volume (3D). In fact, the problem addressed is characterized by high heterogeneity of the images to be segmented, alternating between those without pathology (about 30%, usually concentrated at the apices and bases of the lungs) and others in which lesions involve most of the lungs. Next to the most commonly used 2D metrics, 3D metrics provide comprehensive information on the predictive capabilities of a model.

The 2D metrics we used were the 2D Dice Score (DS), 2D Precision (Pr) and 2D Recall (Re):

(5) $$\mathrm{DS}=\frac{2|P{}_{m}\cap \mathrm{GT}{}_{m}|}{\left|P{}_{m}|+|\mathrm{GT}{}_{m}\right|}=\frac{2\mathrm{TP}}{FP+2TP+FN},$$

(6) $$\mathrm{Pr}=\frac{\left|P{}_{m}\cap \mathrm{GT}{}_{m}\right|}{\left|P{}_{m}\right|}=\frac{\mathrm{TP}}{\mathrm{TP}+\mathrm{FP}},$$

(7) Re=|Pm∩GTm||GTm|=TPTP+FN,

where P_m_ is the Predicted Mask, GT_m_ the Ground Truth Mask, TP the True Positive (i.e., $|P_{m}\cap {\mathrm{GT}}_{m}|$), FP the False Positive, and FN the False Negative.

The number of slices corresponding to anatomical areas above and below the region of interest, i.e., that including the lungs, can vary greatly. For this reason, we expressed the 2D scores also at the patient level, as suggested in [40]. We named these scores the 2D Aggregated Dice score, the 2D Aggregated Precision, and the 2D Aggregated Recall.

The 3D metrics used were the 3D Dice Score (DS), the 3D Precision (Pr), and the 3D Recall (Re). The latter were defined as 2D counterparts, but the predicted and ground truth masks were obtained by combining the 2D masks into a 3D volume.

Due to the high skewness of the distributions of the scores calculated on the predictions (see Section 3 ), none of them follow a Gaussian. Therefore, we reported all the results as medians and the corresponding (25–75%) percentile range.

To compare the predictive capabilities of the models, we took advantage of the fact that the scores were calculated using the same k-fold partitioning, so we could evaluate the results at the patient level. Thus, we used a nonparametric paired test for location, the Wilcoxon paired signed-rank test.

## 3. Results

In this section, we describe the obtained results. In Section 3.1, we show the trends of the loss function during the training of each model. In Section 3.2, we report the values of the metrics chosen for the evaluation of model performance and the statistical analysis performed on them.

### 3.1. Convergence

**Figure 5:**
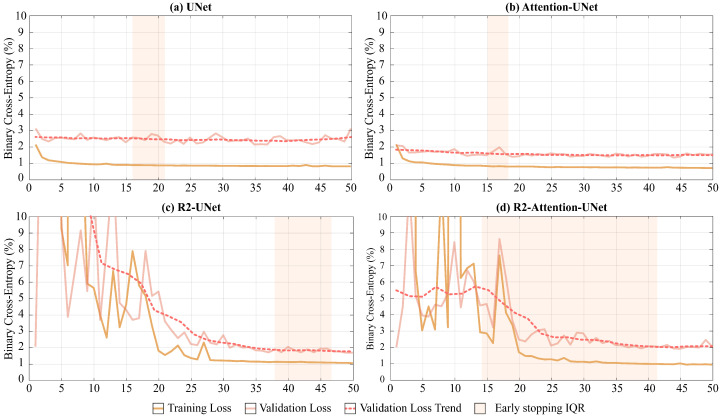
Loss function trends in relation to the number of epochs during the training of the models. The continued dark and light orange line represents, for each epoch, the median loss value over the 5 folds on the training and validation sets, respectively. The dotted line represents the mean trend of the loss function on the validation set for each epoch. Finally, the shaded area in orange represents the range of epochs within which we implemented early stopping as described in Section 2.5.

As shown in Figure 5, UNet and Attention-UNet reached convergence much faster than R2-UNet and R2-Attention UNet. Furthermore, up to the 25th epoch, the latter two models exhibited very unstable loss function trends on both the training and the validation set before reaching convergence.

Regarding the median convergence epoch (i.e., the early stopping epoch), UNet and Attention-UNet show similar behavior since their training stopped at the 15th epoch. Instead, both R2 networks require a higher number of epochs to reach convergence (R2-UNet stopped at the 43th and R2-Attention UNet at the 38th epochs).

Both the increase in the median number of epochs before convergence and the time required to conclude a single epoch for R2 networks lead to significantly different convergence times compared to UNet and Attention-UNet. The minutes needed to end an epoch for UNet, Attention-UNet, R2-UNet, and R2-Attention UNet are, respectively, 12, 13, 23, and 25. Consequently, the median convergence times for UNet and Attention-UNet are comparable (180 and 195 (+8.3% compared to UNet) minutes, respectively). Vice versa, R2-UNet has a median convergence time of 989 min (+449% compared to UNet) while R2-Attention UNet has a median convergence time of 950 min (+427% compared to UNet).

Another major difference between the models is the heterogeneity of performance and early stopping epochs among the different folds. UNet and Attention-UNet have small interquartile ranges (IQR five and three epochs, respectively) proving a very similar behavior among the folds. On the contrary, R2-UNet has an IQR of 9 epochs and R2-Attention-UNet has an IQR of 28 epochs. This discrepancy between folds could reflect a greater need for data for more complex networks. i.e., those with a recurrence mechanism. The total training times are 16 h, 17 h and 30 min, 31 h, and 33 h and 33 min for UNet, Attention-UNet, R2-UNet, and R2-Attention UNet, while the inference times are 21 min, 16 min, 19 min, and 17 min, respectively. In Table 3, the training and inference times are shown, as well as the memory consumption for both RAM and GPU.

### 3.2. Quantitative Results and Comparisons

Performance analysis in the literature is usually performed on the basis of 2D Dice score calculation on all images included in the test set, without grouping the patients included in the test set for each fold. Therefore, for a more consistent comparison with the literature, we first calculated the 2D Dice score on all images in the test set. In Table 4, we report the median and IQR values of the score, thus showing that Attention-UNet reached the maximum value of 81.93%

To more thoroughly evaluate the performance of each model, we next calculated all the metrics described in Section 2.6. We obtained each value in Table 5 by first calculating the medians on the scores of all the slices for each patient in the test set, and then the median on all the values obtained.

In Table 5, Attention-UNet shows the maximum values, for both 2D and 3D Precision, reaching, in the latter case, 92.09%. The values of the Dice score, both 2D and 3D, obtained by Attention-UNet are also the highest among all, the maximum of which is reached in the 3D case, with 79.86%. Regarding the maximum values of 2D and 3D Recall, the former is reached by UNet, the latter by R2-UNet.

**Figure 6:**
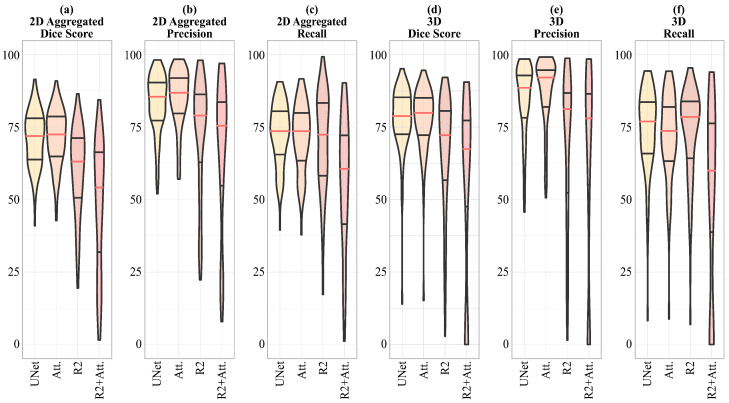
Violin plots representing the median values and the interquartile ranges for 2D aggregated Dice score, Precision and Recall, and 3D aggregated Dice score, Precision, and Recall. Given the significant skewness of the distributions, we indicate that the scores follow a non-normal distribution, thus we choose to apply the Wilcoxon Signed-Rank test for non-parametric data to evaluate the significance of the differences in performance between the models.

In Figure 6, a visual representation of the scores shown in Table 5 is given, emphasizing that for the 2D Dice score, Precision and Recall, and 3D Precision, the dispersion of the R2-UNet and R2-Attention UNet scores is greater than that obtained from the UNet and Attention-UNet models.

To statistically compare differences in model performance and better understand the impact of adding each component and mechanism (e.g., AG for attention, residual connection for recurrence) to the basic UNet architecture, one at a time, we compared the following:

1. UNet and Attention-UNet to evaluate the impact of adding the attention mechanism in the UNet;
2. UNet with R2-UNet to evaluate the impact of adding the recurrent mechanism in the UNet;
3. UNet with R2-Attention to evaluate the impact of adding both the attention and the recurrent mechanism in the UNet;
4. Attention-UNet with R2-Attention UNet to evaluate the effect of adding recurrence in a UNet model that already had attention;
5. R2-UNet with R2-Attention UNet to evaluate the effect of the addition of the attention in a UNet model that already had a recurrence.

Given the non-normality of data distribution shown in Figure 6 where it is evident that the distributions have a significant skewness, we applied the Wilcoxon Signed-Rank test for non-parametric data on the 3D metrics, i.e., on the 3D Dice score, 3D Precision, and 3D Recall.

**Figure 7:**
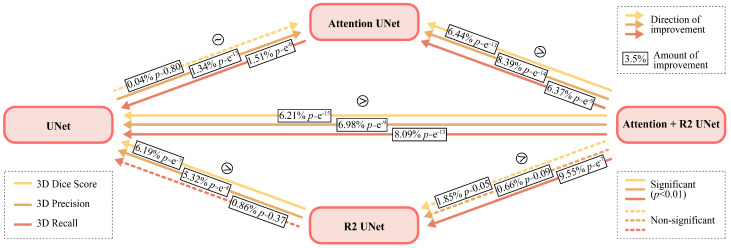
Representation of the results of statistical analysis. The arrows with three different colors indicate the direction of improvement of three different metrics, i.e., yellow for 3D Dice Score, light orange for Precision, and dark orange for Recall. The dotted lines represent non-significant differences. In the boxes placed on each arrow, the difference in percentage is shown, and the *p*-value.

In Figure 7, we represent the results of the analysis mentioned above, also summarized in Table 6.

The results show that the difference in 3D Dice score is not significant between UNet and Attention UNet and between R2-UNet and R2-Attention UNet. Regarding 3D Precision, only the difference between R2-UNet and R2-Attention UNet is not significant. Finally, the difference between UNet and R2-UNet is not significant in terms of 3D Recall.

**Figure 8:**
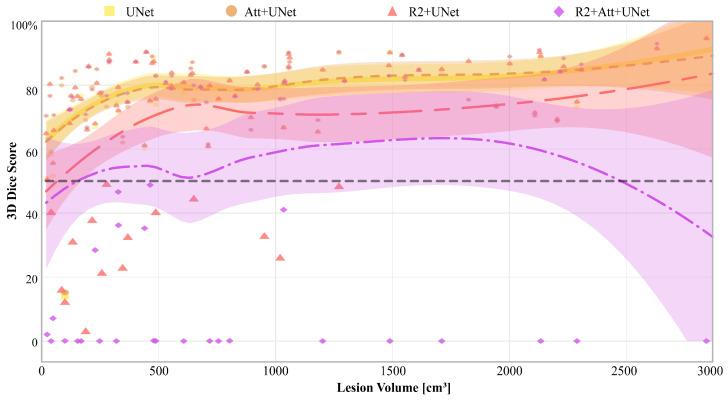
3D Dice score of each model, and on each patient. The yellow squares represent the values obtained from the UNet, the orange circles those from the Attention-UNet, the pink triangles from the R2-UNet, and finally the lilac rhombuses from the R2-Attention UNet. The curves are the Gaussian Process regressions on the 3D Dice score represented as a function of the volume of the disease. The coloured areas visually represent the 95% confidence interval of the respective curve. Finally, the horizontal dotted line reports the threshold of a Dice score equal to 50%, enhanced to better show those patients on which the models performed the worst.

Finally, in Figure 8, we report the 3D Dice scores of each model for each patient, along with the relative Gaussian Process regressions, that are represented as a function of disease volume (expressed in cm^3^), calculated on the ground truth.

It is first inferred that there were some patients who are misclassified by all networks, particularly those with the lowest diseased volumes. Then, UNet and Attention-UNet obtained values greater than 50% on those patients for whom the disease volume was greater than 100 cm^3^. Also, R2-UNet obtained 3D Dice score values greater than 50%, but on patients with a disease volume greater than 1300 cm^3^. Finally, R2-Attention UNet worked generically much worse; in particular, there were numerous patients with 3D Dice scores close to 0%. These cases were mostly concentrated, but not limited, to low-volume lesions, i.e., smaller than 1000 cm^3^.

### 3.3. Ablation Study

To provide a thorough understanding of the functionality and performance of the R2-UNet, Attention-UNet, and R2-Attention UNet architectures, we carried out an ablation study that clarified how individual contributions of each component impact the overall performance.

To perform the ablation study, all models were evaluated by removing the peculiar components, namely recurrent, residual blocks for R2-UNet, attention gates for Attention-UNet and recurrent, residual blocks with attention gates for R2-Attention UNet, one at a time.

More precisely, for an initial assessment in the case of R2-UNet, we placed the recurrent, residual blocks R1 and R2 in Stages (iv) and (v) of Figure 4. Then, we removed the residual block in Stage (v), evaluating the performance with only the recurrent, residual block R1 in Stage (iv).

Concurrently, we adopted a similar approach to the Attention-UNet model by first training and testing the network with AGs A1 and A2 on Stages (iv) and (v) of Figure 4, and then only with A1 on Stage (iv).

Finally, for R2-Attention-UNet, we first added two recurrent, residual blocks R1 and R2 with AGs A1 and A2 in Stages (iv) and (v), and then we put only the recurrent and residual block R1 in Stage (iv), with one AG A1.

We trained and tested each model on the entire dataset, without performing five-fold cross-validation, and we presented the numerical values of the 2D Dice score for each model in Table 7.

## 4. Discussion and Conclusions

In this work, we evaluated the performance of four distinct UNet-based Convolutional Neural Networks (CNNs), namely UNet, R2-UNet, Attention-UNet, and R2-Attention UNet, using the novel OPTIMISED project dataset comprising 90 COVID-19 patients. We investigated whether integrating advanced mechanisms, such as attention and recurrence, could enhance the accuracy of segmenting the typical disease infections. Thus, the considered CNNs differed only by the presence of attention and recurrence and were invariant for all other hyperparameters (e.g., number of trainable parameters, number of layers, pooling strategies). In order to compare the performance at the patient level, the models were trained using the same five-fold cross-validation scheme. Evaluations were based on a combination of traditional (e.g., 2D Dice score) and ad hoc segmentation scores (e.g., 3D Dice score, aggregated 2D scores) to mitigate the effects of patient variability.

This rigorously structured analysis offered us an in-depth view of the strengths and weaknesses of each model. First of all, Attention-UNet emerges as the best performing model for the task of binary segmentation of COVID-19 infections. Remarkably, it achieved superior performance, reaching a value of over 80% for the 2D Dice score, with a convergence time of approximately 3 h. On the contrary, the recurrence mechanism seems to deteriorate the performance in terms of each of the chosen metrics (reduction from −7% to −21%). From the point of view of convergence time and computational resources required, recurrence has disproportionate computational loads compared to the results obtained, leading not only to an increase of about +400% in the time required for convergence compared to UNet but also to a memory load exceeding 11 GB. This imbalance underscores the inefficiency of incorporating recurrent mechanisms for the COVID-19 binary segmentation task, revealing the need for more streamlined and efficient approaches.

**Figure 9:**
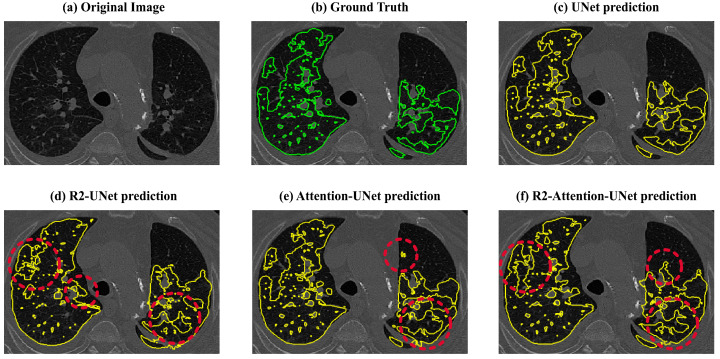
Visual comparisons between the ground truths (green) and the predictions (yellow) of UNet, R2-UNet, Attention-UNet, and R2 Attention-UNet. The red circles contain the areas where the models overestimated the disease.

Lastly, the recurrence mechanism leads to an overestimation of the amount of disease (also visible in Figure 9). This overestimation significantly impacts the Dice scores, reducing them to below 50% for both R2 and R2-Attention UNet, especially when the amount of the disease is low. This tendency to overestimate disease is further evident in Precision values, both 2D and 3D, derived from R2 and R2-Attention UNet, which fall below those obtained by UNet and Attention-UNet.

Furthermore, in our comprehensive ablation study, we systematically investigated the impact of attention gate positioning within the architecture of an Attention U-Net for the analyzed task. Our findings reveal a crucial insight into the optimal configuration of attention mechanisms. Remarkably, the results demonstrate that superior performance is achieved when attention gates are placed at every stage in the decoding path. This placement ensures that the network effectively captures and leverages long-range dependencies, thereby enhancing the model’s overall performance. On the contrary, examination of recurrent mechanisms elucidated a distinct phenomenon. Specifically, the inclusion of attention gates at each stage within the recurrent mechanism in R2-Attention UNet exacerbates performance degradation. This counterintuitive observation underscores the nuanced interplay between attention mechanisms and recurrent architectures, highlighting the importance of thoughtful design choices in the pursuit of optimal model performance.

The better performance of attention with respect to recurrence in the analyzed task may be due to the fact that attention can adaptively weigh different parts of the image based on their relevance to the task while capturing complex patterns and relationships within it. On the contrary, recurrence may struggle to capture complex spatial dependencies and may require more complex architectures than UNet to model intricate patterns effectively, especially in COVID-19 segmentation since the disease is sometimes randomly spread over the entire image. From the point of view of computational load, since recurrence operates sequentially, the processing of one part of the image at a time may require longer training and inference times. Moreover, recurrent mechanisms may need to store information about the entire sequence, leading to higher memory requirements.

To summarize, for this task, these results emphasize that it is effective to adapt a simple framework to the size and the nature of the data and to avoid more complex architectures.

However, a limitation of our work is the fact that the analysis was applied only to the OPTIMISED project dataset, which currently includes a total of 90 patients. The lack of an external independent test set was addressed by the use of a rigorous k-fold cross-validation, which can provide an estimate of the generalization capability of the models. A second minor limitation regards the use of binary segmentation models which only a focus on distinguishing diseased tissues from healthy ones. Whereas this classification approach is sufficient to correctly define diagnosis and prognosis, adding a multi-class strategy to differentiate between GGO and consolidations could improve the assessment of disease severity more than binary quantification of disease. Indeed, in clinical practice, radiologists need to quantify each individual pattern because, on the one hand, the presence of GGO is often associated with early or mild disease, and on the other hand, consolidations may indicate more severe lung tissue involvement such that the lungs may be irreversibly compromised.

Our upcoming study will center on the integration of additional data and an external validation procedure into a unique framework based on Attention-UNet, with optimal capability to generalize on unseen data, starting from the limits and outcomes produced in this work. In fact, we will add more sophisticated components to Attention-UNet’s architecture to enable the transition from binary to multi-class segmentation of COVID-19 infections, as we discovered that the attention mechanism performs better than the others for the binary segmentation of COVID-19-related infections. Moreover, we will define ad hoc GANs to generate more synthetic data in an effort to increase data variability. 

## Figures and Tables

**Table 1:** Main information about literature papers on COVID-19 segmentation.

Reference	Dataset: n. Patients (n. Images)	Cross-Validation	Results (Dice Score)
[13]	Inf-Net: >40 (100) Semi-Inf-Net: 20 (1600)	No	Inf-Net: 68.2% Semi-Inf-Net: 73.9%
[14]	60 (4630)	No	68.43%
[15]	20 (1800+)	No	80.34%
[16]	40 (100)	N	92.46%
[17]	20	No	82%
[18]	49 (929)	No	86%
[19]	>661 (295)	No	75.4% (first ranked)
[26]	>69 (1838)	No	82%
[27]	69 (473)	No	83.1%
[28]	38 (1745)	No	72.98%
[29]	>69 (3000 data augmentation)	Yes	76%
[30]	219 (5199)	No	77.60%
[31]	>40 (1963)	Yes	86.96%
[32]	>40 (100)	Yes	77.15%
[33]	60 (110)	No	93.4%
[34]	60 (600 data augmentation)	Yes	94%
[35]	>230 (32,714)	Yes	77.6%

**Table 2:** OPTIMIZED Dataset characteristics.

Characteristics	Median [IQR]
Number of slices	296 [279–315]
Number of diseased slices	218 [203–246]
Healthy slices over diseased slices (%)	45.36% [19.31–48.28%]
Ground truth area (mm^2^)	428.93 [4.33–25.81]
Ground truth volume (mm^3^)	640,852.6 [262,534.03–1,253,504.56]
Pixel spacing (mm)	0.68 [0.62–0.72]
Slice thickness (mm)	1.44 [1.34–1.50]
Slice dimensions	512×512

**Table 3:** Training and inference times and memory usage (in terms of RAM and maximum GPU consumption) for each model.

	UNet	Attention-UNet	R2-UNet	R2-Att UNet
Training Time	16 h	17 h 30 min	31 h	33 h 30 min
Inference Time	21 min	16 min	19 min	17 min
Trainable parameters	1,946,305	1,978,900	5,973,889	6,006,484
RAM consumption	3.16 GB	3.99 GB	11.67 GB	12.50 GB
Maximum GPU consumption	1.28 GB	2.24 GB	5.20 GB	6.40 GB

**Table 4:** Median values and IQR of 2D Dice score computed on all the images of the test set. In green is the maximum value.

	2D Dice Score
UNet	81.88% [63.73–91.63%]
Attention-UNet	81.93% [64.17–91.65%]
R2-UNet	72.38% [32.3–87.05%]
R2-Attention UNet	60.40% [0–84.46%]

**Table 5:** Values of metrics for evaluating the performances of the models obtained by grouping the test set per patient at each fold. Highlighted in green are the maximum values for each metric, with an underline for the highest value of all metrics. All the values are expressed as median [IQR].

	Dice Score 2D	Precision 2D	Recall 2D	Dice Score 3D	Precision 3D	Recall 3D
UNet	72.05 [64.23–78.15]	85.45 [78.55–90.63]	**73.59** [65.77–80.97]	78.77 [73.20–85.27]	88.52 [80.89–94.66]	76.95 [67.60–84.42]
Att UNet	**72.43** [65.25–78.08]	**86.82** [80.46–93.06]	73.52 [65.11–80.20]	**79.86** [73.35–85.62]	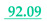 [83.25–95.99]	73.64 [64.45–84.05]
R2 UNet	63.11 [50.97–71.13]	79.00 [63.90–86.98]	72.40 [59.48–84.58]	72.27 [59.08–82.10]	81.12 [54.87–89.66]	**78.47** [64.76–83.65]
Att+R2 UNet	54.17 [29.41–68.39]	75.47 [56.51–85.65]	60.60 [43.47–73.17]	67.42 [37.42–81.28]	78.03 [49.93–90.16]	59.96 [27.27–79.19]

**Table 6:** Results of statistical analysis. The values in each cell represent the percentage increase in the metric. In red are highlighted the values with no significance.

	3D Dice Score	3D Precision	3D Recall
UNet vs. Attention-UNet (1)	0.04%	1.34%	1.51%
UNet vs. R2-UNet (2)	6.19%	5.32%	0.86%
UNet vs. R2-Attention UNet (3)	6.21%	6.98%	8.09%
Attention-UNet vs. R2-Attention UNet (4)	6.44%	8.39%	6.37%
R2-UNet vs. R2-Attention UNet (5)	1.85%	0.66%	9.55%

**Table 7:** Median values and interquartile ranges of 2D Dice score of each model under ablation study. The maximum values for each model are highlighted in green.

Model	2D Dice Score
UNet	79.51% [63.31–91.33%]
Attention-UNet with A1	76.43% [41.80–87.81%]
Attention-UNet with A1 and A2	82.20% [58.59–91.90%]
Attention-UNet with A1, A2, and A3	83.14% [60.99–91.82%]
R2-UNet with R1	68.85% [59.37–90.95%]
R2-UNet with R1 and R2	69.43% [21.66–83.79%]
R2-UNet with R1, R2 and R3	73.61% [28.98–87.33%]
R2-Attention UNet with R1&A1	72.38% [28.82–85.77%]
R2-Attention UNet with R1&A1 and R2&A2	70.51% [64.37–92.34%]
R2-Attention UNet with R1&A1, R2&A2, and R3&A3	57.17% [23.09–83.96%]

## Data Availability

The data used in this work are unavailable due to privacy or ethical restrictions.

## Supplementary Materials

The following supporting information can be downloaded at: https://www.mdpi.com/article/10.3390/jimaging9120283/s1, Table S1: Transformation functions layer by layer of the UNet architecture; Table S2: Transformation functions layer by layer of the R2-UNet architecture; Table S3: Transformation functions layer by layer of the Attention-UNet architecture; Table S4: Transformation functions layer by layer of the R2-Attention UNet architecture.
