# Supplementary material for: Enhancing COVID-19 CT Image Segmentation: A Comparative Study of Attention and Recurrence in UNet Models
Source: J Imaging. 2023 Dec 18;9(12):283. doi: 10.3390/jimaging9120283 (PMC10744014; doi:10.3390/jimaging9120283)
Supplement: Supplementary file 1 [file jimaging-09-00283-s001.zip › jimaging-2694529-supplementary.pdf]

UNet

Layer #	Name	Type	Shape
0	input_1	InputLayer	[(None, 1, 512, 512)]
1	conv2d	Conv2D	(None, 32, 512, 512)
2	dropout	Dropout	(None, 32, 512, 512)
3	conv2d_1	Conv2D	(None, 32, 512, 512)
4	max_pooling2d	MaxPooling2D	(None, 32, 256, 256)
5	conv2d_2	Conv2D	(None, 64, 256, 256)
6	dropout_1	Dropout	(None, 64, 256, 256)
7	conv2d_3	Conv2D	(None, 64, 256, 256)
8	max_pooling2d_1	MaxPooling2D	(None, 64, 128, 128)
9	conv2d_4	Conv2D	(None, 128, 128, 128)
10	dropout_2	Dropout	(None, 128, 128, 128)
11	conv2d_5	Conv2D	(None, 128, 128, 128)
12	max_pooling2d_2	MaxPooling2D	(None, 128, 64, 64)
13	conv2d_6	Conv2D	(None, 256, 64, 64)
14	dropout_3	Dropout	(None, 256, 64, 64)
15	conv2d_7	Conv2D	(None, 256, 64, 64)
16	up_sampling2d	UpSampling2D	(None, 256, 128, 128)
17	concatenate	Concatenate	(None, 384, 128, 128)
18	conv2d_8	Conv2D	(None, 128, 128, 128)
19	dropout_4	Dropout	(None, 128, 128, 128)
20	conv2d_9	Conv2D	(None, 128, 128, 128)
21	up_sampling2d_1	UpSampling2D	(None, 128, 256, 256)
22	concatenate_1	Concatenate	(None, 192, 256, 256)
23	conv2d_10	Conv2D	(None, 64, 256, 256)
24	dropout_5	Dropout	(None, 64, 256, 256)
25	conv2d_11	Conv2D	(None, 64, 256, 256)
26	up_sampling2d_2	UpSampling2D	(None, 64, 512, 512)
27	concatenate_2	Concatenate	(None, 96, 512, 512)
28	conv2d_12	Conv2D	(None, 32, 512, 512)
29	dropout_6	Dropout	(None, 32, 512, 512)
30	conv2d_13	Conv2D	(None, 32, 512, 512)
31	conv2d_14	Conv2D	(None, 1, 512, 512)
32	activation	Activation	(None, 1, 512, 512)

Table S1: Transformation functions layer by layer of the UNet architecture

R2-UNet

Layer #	Name	Type	Shape
0	input_2	InputLayer	[(None, 1, 512, 512)]
1	conv2d_15	Conv2D	(None, 32, 512, 512)
2	conv2d_16	Conv2D	(None, 32, 512, 512)
3	activation_1	Activation	(None, 32, 512, 512)
4	add	Add	(None, 32, 512, 512)
5	conv2d_17	Conv2D	(None, 32, 512, 512)
6	activation_2	Activation	(None, 32, 512, 512)
7	add_1	Add	(None, 32, 512, 512)
8	conv2d_18	Conv2D	(None, 32, 512, 512)
9	activation_3	Activation	(None, 32, 512, 512)
10	conv2d_19	Conv2D	(None, 32, 512, 512)
11	activation_4	Activation	(None, 32, 512, 512)
12	add_2	Add	(None, 32, 512, 512)
13	conv2d_20	Conv2D	(None, 32, 512, 512)
14	activation_5	Activation	(None, 32, 512, 512)
15	add_3	Add	(None, 32, 512, 512)
16	conv2d_21	Conv2D	(None, 32, 512, 512)
17	activation_6	Activation	(None, 32, 512, 512)
18	add_4	Add	(None, 32, 512, 512)
19	max_pooling2d_3	MaxPooling2D	(None, 32, 256, 256)
20	conv2d_22	Conv2D	(None, 64, 256, 256)
21	conv2d_23	Conv2D	(None, 64, 256, 256)
22	activation_7	Activation	(None, 64, 256, 256)
23	add_5	Add	(None, 64, 256, 256)
24	conv2d_24	Conv2D	(None, 64, 256, 256)
25	activation_8	Activation	(None, 64, 256, 256)
26	add_6	Add	(None, 64, 256, 256)
27	conv2d_25	Conv2D	(None, 64, 256, 256)
28	activation_9	Activation	(None, 64, 256, 256)
29	conv2d_26	Conv2D	(None, 64, 256, 256)
30	activation_10	Activation	(None, 64, 256, 256)
31	add_7	Add	(None, 64, 256, 256)
32	conv2d_27	Conv2D	(None, 64, 256, 256)
33	activation_11	Activation	(None, 64, 256, 256)
34	add_8	Add	(None, 64, 256, 256)
35	conv2d_28	Conv2D	(None, 64, 256, 256)
36	activation_12	Activation	(None, 64, 256, 256)
37	add_9	Add	(None, 64, 256, 256)
38	max_pooling2d_4	MaxPooling2D	(None, 64, 128, 128)
39	conv2d_29	Conv2D	(None, 128, 128, 128)
40	conv2d_30	Conv2D	(None, 128, 128, 128)
41	activation_13	Activation	(None, 128, 128, 128)
42	add_10	Add	(None, 128, 128, 128)
43	conv2d_31	Conv2D	(None, 128, 128, 128)
44	activation_14	Activation	(None, 128, 128, 128)
45	add_11	Add	(None, 128, 128, 128)
46	conv2d_32	Conv2D	(None, 128, 128, 128)
47	activation_15	Activation	(None, 128, 128, 128)

48	conv2d_33	Conv2D	(None, 128, 128, 128)
49	activation_16	Activation	(None, 128, 128, 128)
50	add_12	Add	(None, 128, 128, 128)
51	conv2d_34	Conv2D	(None, 128, 128, 128)
52	activation_17	Activation	(None, 128, 128, 128)
53	add_13	Add	(None, 128, 128, 128)
54	conv2d_35	Conv2D	(None, 128, 128, 128)
55	activation_18	Activation	(None, 128, 128, 128)
56	add_14	Add	(None, 128, 128, 128)
57	max_pooling2d_5	MaxPooling2D	(None, 128, 64, 64)
58	conv2d_36	Conv2D	(None, 256, 64, 64)
59	conv2d_37	Conv2D	(None, 256, 64, 64)
60	activation_19	Activation	(None, 256, 64, 64)
61	add_15	Add	(None, 256, 64, 64)
62	conv2d_38	Conv2D	(None, 256, 64, 64)
63	activation_20	Activation	(None, 256, 64, 64)
64	add_16	Add	(None, 256, 64, 64)
65	conv2d_39	Conv2D	(None, 256, 64, 64)
66	activation_21	Activation	(None, 256, 64, 64)
67	conv2d_40	Conv2D	(None, 256, 64, 64)
68	activation_22	Activation	(None, 256, 64, 64)
69	add_17	Add	(None, 256, 64, 64)
70	conv2d_41	Conv2D	(None, 256, 64, 64)
71	activation_23	Activation	(None, 256, 64, 64)
72	add_18	Add	(None, 256, 64, 64)
73	conv2d_42	Conv2D	(None, 256, 64, 64)
74	activation_24	Activation	(None, 256, 64, 64)
75	add_19	Add	(None, 256, 64, 64)
76	up_sampling2d_3	UpSampling2D	(None, 256, 128, 128)
77	lambda	Lambda	(None, 384, 128, 128)
78	conv2d_43	Conv2D	(None, 128, 128, 128)
79	conv2d_44	Conv2D	(None, 128, 128, 128)
80	activation_25	Activation	(None, 128, 128, 128)
81	add_20	Add	(None, 128, 128, 128)
82	conv2d_45	Conv2D	(None, 128, 128, 128)
83	activation_26	Activation	(None, 128, 128, 128)
84	add_21	Add	(None, 128, 128, 128)
85	conv2d_46	Conv2D	(None, 128, 128, 128)
86	activation_27	Activation	(None, 128, 128, 128)
87	conv2d_47	Conv2D	(None, 128, 128, 128)
88	activation_28	Activation	(None, 128, 128, 128)
89	add_22	Add	(None, 128, 128, 128)
90	conv2d_48	Conv2D	(None, 128, 128, 128)
91	activation_29	Activation	(None, 128, 128, 128)
92	add_23	Add	(None, 128, 128, 128)
93	conv2d_49	Conv2D	(None, 128, 128, 128)
94	activation_30	Activation	(None, 128, 128, 128)
95	add_24	Add	(None, 128, 128, 128)
96	up_sampling2d_4	UpSampling2D	(None, 128, 256, 256)
97	lambda_1	Lambda	(None, 192, 256, 256)

98	conv2d_50	Conv2D	(None, 64, 256, 256)
99	conv2d_51	Conv2D	(None, 64, 256, 256)
100	activation_31	Activation	(None, 64, 256, 256)
101	add_25	Add	(None, 64, 256, 256)
102	conv2d_52	Conv2D	(None, 64, 256, 256)
103	activation_32	Activation	(None, 64, 256, 256)
104	add_26	Add	(None, 64, 256, 256)
105	conv2d_53	Conv2D	(None, 64, 256, 256)
106	activation_33	Activation	(None, 64, 256, 256)
107	conv2d_54	Conv2D	(None, 64, 256, 256)
108	activation_34	Activation	(None, 64, 256, 256)
109	add_27	Add	(None, 64, 256, 256)
110	conv2d_55	Conv2D	(None, 64, 256, 256)
111	activation_35	Activation	(None, 64, 256, 256)
112	add_28	Add	(None, 64, 256, 256)
113	conv2d_56	Conv2D	(None, 64, 256, 256)
114	activation_36	Activation	(None, 64, 256, 256)
115	add_29	Add	(None, 64, 256, 256)
116	up_sampling2d_5	UpSampling2D	(None, 64, 512, 512)
117	lambda_2	Lambda	(None, 96, 512, 512)
118	conv2d_57	Conv2D	(None, 32, 512, 512)
119	conv2d_58	Conv2D	(None, 32, 512, 512)
120	activation_37	Activation	(None, 32, 512, 512)
121	add_30	Add	(None, 32, 512, 512)
122	conv2d_59	Conv2D	(None, 32, 512, 512)
123	activation_38	Activation	(None, 32, 512, 512)
124	add_31	Add	(None, 32, 512, 512)
125	conv2d_60	Conv2D	(None, 32, 512, 512)
126	activation_39	Activation	(None, 32, 512, 512)
127	conv2d_61	Conv2D	(None, 32, 512, 512)
128	activation_40	Activation	(None, 32, 512, 512)
129	add_32	Add	(None, 32, 512, 512)
130	conv2d_62	Conv2D	(None, 32, 512, 512)
131	activation_41	Activation	(None, 32, 512, 512)
132	add_33	Add	(None, 32, 512, 512)
133	conv2d_63	Conv2D	(None, 32, 512, 512)
134	activation_42	Activation	(None, 32, 512, 512)
135	add_34	Add	(None, 32, 512, 512)
136	conv2d_64	Conv2D	(None, 1, 512, 512)
137	activation_43	Activation	(None, 1, 512, 512)

Table S2: Transformation functions layer by layer of the R2-UNet architecture

Attention-UNet

Layer #	Name	Type	Shape
0	input_1	InputLayer	[(None, 1, 512, 512)]
1	conv2d	Conv2D	(None, 32, 512, 512)
2	dropout	Dropout	(None, 32, 512, 512)
3	conv2d_1	Conv2D	(None, 32, 512, 512)
4	max_pooling2d	MaxPooling2D	(None, 32, 256, 256)
5	conv2d_2	Conv2D	(None, 64, 256, 256)
6	dropout_1	Dropout	(None, 64, 256, 256)
7	conv2d_3	Conv2D	(None, 64, 256, 256)
8	max_pooling2d_1	MaxPooling2D	(None, 64, 128, 128)
9	conv2d_4	Conv2D	(None, 128, 128, 128)
10	dropout_2	Dropout	(None, 128, 128, 128)
11	conv2d_5	Conv2D	(None, 128, 128, 128)
12	max_pooling2d_2	MaxPooling2D	(None, 128, 64, 64)
13	conv2d_6	Conv2D	(None, 256, 64, 64)
14	dropout_3	Dropout	(None, 256, 64, 64)
15	conv2d_7	Conv2D	(None, 256, 64, 64)
16	up_sampling2d	UpSampling2D	(None, 256, 128, 128)
17	conv2d_8	Conv2D	(None, 64, 128, 128)
18	conv2d_9	Conv2D	(None, 64, 128, 128)
19	add	Add	(None, 64, 128, 128)
20	activation	Activation	(None, 64, 128, 128)
21	conv2d_10	Conv2D	(None, 1, 128, 128)
22	activation_1	Activation	(None, 1, 128, 128)
23	multiply	Multiply	(None, 128, 128, 128)
24	lambda	Lambda	(None, 384, 128, 128)
25	conv2d_11	Conv2D	(None, 128, 128, 128)
26	dropout_4	Dropout	(None, 128, 128, 128)
27	conv2d_12	Conv2D	(None, 128, 128, 128)
28	up_sampling2d_1	UpSampling2D	(None, 128, 256, 256)
29	conv2d_13	Conv2D	(None, 32, 256, 256)
30	conv2d_14	Conv2D	(None, 32, 256, 256)
31	add_1	Add	(None, 32, 256, 256)
32	activation_2	Activation	(None, 32, 256, 256)
33	conv2d_15	Conv2D	(None, 1, 256, 256)
34	activation_3	Activation	(None, 1, 256, 256)
35	multiply_1	Multiply	(None, 64, 256, 256)
36	lambda_1	Lambda	(None, 192, 256, 256)
37	conv2d_16	Conv2D	(None, 64, 256, 256)
38	dropout_5	Dropout	(None, 64, 256, 256)
39	conv2d_17	Conv2D	(None, 64, 256, 256)
40	up_sampling2d_2	UpSampling2D	(None, 64, 512, 512)
41	conv2d_18	Conv2D	(None, 16, 512, 512)
42	conv2d_19	Conv2D	(None, 16, 512, 512)
43	add_2	Add	(None, 16, 512, 512)
44	activation_4	Activation	(None, 16, 512, 512)
45	conv2d_20	Conv2D	(None, 1, 512, 512)
46	activation_5	Activation	(None, 1, 512, 512)
47	multiply_2	Multiply	(None, 32, 512, 512)

48	lambda_2	Lambda	(None, 96, 512, 512)
49	conv2d_21	Conv2D	(None, 32, 512, 512)
50	dropout_6	Dropout	(None, 32, 512, 512)
51	conv2d_22	Conv2D	(None, 32, 512, 512)
52	conv2d_23	Conv2D	(None, 1, 512, 512)
53	activation_6	Activation	(None, 1, 512, 512)

Table S3: Transformation functions layer by layer of the Attention-UNet architecture

R2-Attention UNet

Layer #	Name	Type	Shape
0	input_3	InputLayer	[(None, 1, 512, 512)]
1	conv2d_65	Conv2D	(None, 32, 512, 512)
2	conv2d_66	Conv2D	(None, 32, 512, 512)
3	activation_44	Activation	(None, 32, 512, 512)
4	add_35	Add	(None, 32, 512, 512)
5	conv2d_67	Conv2D	(None, 32, 512, 512)
6	activation_45	Activation	(None, 32, 512, 512)
7	add_36	Add	(None, 32, 512, 512)
8	conv2d_68	Conv2D	(None, 32, 512, 512)
9	activation_46	Activation	(None, 32, 512, 512)
10	conv2d_69	Conv2D	(None, 32, 512, 512)
11	activation_47	Activation	(None, 32, 512, 512)
12	add_37	Add	(None, 32, 512, 512)
13	conv2d_70	Conv2D	(None, 32, 512, 512)
14	activation_48	Activation	(None, 32, 512, 512)
15	add_38	Add	(None, 32, 512, 512)
16	conv2d_71	Conv2D	(None, 32, 512, 512)
17	activation_49	Activation	(None, 32, 512, 512)
18	add_39	Add	(None, 32, 512, 512)
19	max_pooling2d_6	MaxPooling2D	(None, 32, 256, 256)
20	conv2d_72	Conv2D	(None, 64, 256, 256)
21	conv2d_73	Conv2D	(None, 64, 256, 256)
22	activation_50	Activation	(None, 64, 256, 256)
23	add_40	Add	(None, 64, 256, 256)
24	conv2d_74	Conv2D	(None, 64, 256, 256)
25	activation_51	Activation	(None, 64, 256, 256)
26	add_41	Add	(None, 64, 256, 256)
27	conv2d_75	Conv2D	(None, 64, 256, 256)
28	activation_52	Activation	(None, 64, 256, 256)
29	conv2d_76	Conv2D	(None, 64, 256, 256)
30	activation_53	Activation	(None, 64, 256, 256)
31	add_42	Add	(None, 64, 256, 256)
32	conv2d_77	Conv2D	(None, 64, 256, 256)
33	activation_54	Activation	(None, 64, 256, 256)
34	add_43	Add	(None, 64, 256, 256)
35	conv2d_78	Conv2D	(None, 64, 256, 256)
36	activation_55	Activation	(None, 64, 256, 256)
37	add_44	Add	(None, 64, 256, 256)
38	max_pooling2d_7	MaxPooling2D	(None, 64, 128, 128)
39	conv2d_79	Conv2D	(None, 128, 128, 128)
40	conv2d_80	Conv2D	(None, 128, 128, 128)
41	activation_56	Activation	(None, 128, 128, 128)
42	add_45	Add	(None, 128, 128, 128)
43	conv2d_81	Conv2D	(None, 128, 128, 128)
44	activation_57	Activation	(None, 128, 128, 128)
45	add_46	Add	(None, 128, 128, 128)
46	conv2d_82	Conv2D	(None, 128, 128, 128)
47	activation_58	Activation	(None, 128, 128, 128)

48	conv2d_83	Conv2D	(None, 128, 128, 128)
49	activation_59	Activation	(None, 128, 128, 128)
50	add_47	Add	(None, 128, 128, 128)
51	conv2d_84	Conv2D	(None, 128, 128, 128)
52	activation_60	Activation	(None, 128, 128, 128)
53	add_48	Add	(None, 128, 128, 128)
54	conv2d_85	Conv2D	(None, 128, 128, 128)
55	activation_61	Activation	(None, 128, 128, 128)
56	add_49	Add	(None, 128, 128, 128)
57	max_pooling2d_8	MaxPooling2D	(None, 128, 64, 64)
58	conv2d_86	Conv2D	(None, 256, 64, 64)
59	conv2d_87	Conv2D	(None, 256, 64, 64)
60	activation_62	Activation	(None, 256, 64, 64)
61	add_50	Add	(None, 256, 64, 64)
62	conv2d_88	Conv2D	(None, 256, 64, 64)
63	activation_63	Activation	(None, 256, 64, 64)
64	add_51	Add	(None, 256, 64, 64)
65	conv2d_89	Conv2D	(None, 256, 64, 64)
66	activation_64	Activation	(None, 256, 64, 64)
67	conv2d_90	Conv2D	(None, 256, 64, 64)
68	activation_65	Activation	(None, 256, 64, 64)
69	add_52	Add	(None, 256, 64, 64)
70	conv2d_91	Conv2D	(None, 256, 64, 64)
71	activation_66	Activation	(None, 256, 64, 64)
72	add_53	Add	(None, 256, 64, 64)
73	conv2d_92	Conv2D	(None, 256, 64, 64)
74	activation_67	Activation	(None, 256, 64, 64)
75	add_54	Add	(None, 256, 64, 64)
76	up_sampling2d_6	UpSampling2D	(None, 256, 128, 128)
77	conv2d_93	Conv2D	(None, 64, 128, 128)
78	conv2d_94	Conv2D	(None, 64, 128, 128)
79	add_55	Add	(None, 64, 128, 128)
80	activation_68	Activation	(None, 64, 128, 128)
81	conv2d_95	Conv2D	(None, 1, 128, 128)
82	activation_69	Activation	(None, 1, 128, 128)
83	multiply	Multiply	(None, 128, 128, 128)
84	lambda_3	Lambda	(None, 384, 128, 128)
85	conv2d_96	Conv2D	(None, 128, 128, 128)
86	conv2d_97	Conv2D	(None, 128, 128, 128)
87	activation_70	Activation	(None, 128, 128, 128)
88	add_56	Add	(None, 128, 128, 128)
89	conv2d_98	Conv2D	(None, 128, 128, 128)
90	activation_71	Activation	(None, 128, 128, 128)
91	add_57	Add	(None, 128, 128, 128)
92	conv2d_99	Conv2D	(None, 128, 128, 128)
93	activation_72	Activation	(None, 128, 128, 128)
94	conv2d_100	Conv2D	(None, 128, 128, 128)
95	activation_73	Activation	(None, 128, 128, 128)
96	add_58	Add	(None, 128, 128, 128)
97	conv2d_101	Conv2D	(None, 128, 128, 128)

98	activation_74	Activation	(None, 128, 128, 128)
99	add_59	Add	(None, 128, 128, 128)
100	conv2d_102	Conv2D	(None, 128, 128, 128)
101	activation_75	Activation	(None, 128, 128, 128)
102	add_60	Add	(None, 128, 128, 128)
103	up_sampling2d_7	UpSampling2D	(None, 128, 256, 256)
104	conv2d_103	Conv2D	(None, 32, 256, 256)
105	conv2d_104	Conv2D	(None, 32, 256, 256)
106	add_61	Add	(None, 32, 256, 256)
107	activation_76	Activation	(None, 32, 256, 256)
108	conv2d_105	Conv2D	(None, 1, 256, 256)
109	activation_77	Activation	(None, 1, 256, 256)
110	multiply_1	Multiply	(None, 64, 256, 256)
111	lambda_4	Lambda	(None, 192, 256, 256)
112	conv2d_106	Conv2D	(None, 64, 256, 256)
113	conv2d_107	Conv2D	(None, 64, 256, 256)
114	activation_78	Activation	(None, 64, 256, 256)
115	add_62	Add	(None, 64, 256, 256)
116	conv2d_108	Conv2D	(None, 64, 256, 256)
117	activation_79	Activation	(None, 64, 256, 256)
118	add_63	Add	(None, 64, 256, 256)
119	conv2d_109	Conv2D	(None, 64, 256, 256)
120	activation_80	Activation	(None, 64, 256, 256)
121	conv2d_110	Conv2D	(None, 64, 256, 256)
122	activation_81	Activation	(None, 64, 256, 256)
123	add_64	Add	(None, 64, 256, 256)
124	conv2d_111	Conv2D	(None, 64, 256, 256)
125	activation_82	Activation	(None, 64, 256, 256)
126	add_65	Add	(None, 64, 256, 256)
127	conv2d_112	Conv2D	(None, 64, 256, 256)
128	activation_83	Activation	(None, 64, 256, 256)
129	add_66	Add	(None, 64, 256, 256)
130	up_sampling2d_8	UpSampling2D	(None, 64, 512, 512)
131	conv2d_113	Conv2D	(None, 16, 512, 512)
132	conv2d_114	Conv2D	(None, 16, 512, 512)
133	add_67	Add	(None, 16, 512, 512)
134	activation_84	Activation	(None, 16, 512, 512)
135	conv2d_115	Conv2D	(None, 1, 512, 512)
136	activation_85	Activation	(None, 1, 512, 512)
137	multiply_2	Multiply	(None, 32, 512, 512)
138	lambda_5	Lambda	(None, 96, 512, 512)
139	conv2d_116	Conv2D	(None, 32, 512, 512)
140	conv2d_117	Conv2D	(None, 32, 512, 512)
141	activation_86	Activation	(None, 32, 512, 512)
142	add_68	Add	(None, 32, 512, 512)
143	conv2d_118	Conv2D	(None, 32, 512, 512)
144	activation_87	Activation	(None, 32, 512, 512)
145	add_69	Add	(None, 32, 512, 512)
146	conv2d_119	Conv2D	(None, 32, 512, 512)
147	activation_88	Activation	(None, 32, 512, 512)

148	conv2d_120	Conv2D	(None, 32, 512, 512)
149	activation_89	Activation	(None, 32, 512, 512)
150	add_70	Add	(None, 32, 512, 512)
151	conv2d_121	Conv2D	(None, 32, 512, 512)
152	activation_90	Activation	(None, 32, 512, 512)
153	add_71	Add	(None, 32, 512, 512)
154	conv2d_122	Conv2D	(None, 32, 512, 512)
155	activation_91	Activation	(None, 32, 512, 512)
156	add_72	Add	(None, 32, 512, 512)
157	conv2d_123	Conv2D	(None, 1, 512, 512)
158	activation_92	Activation	(None, 1, 512, 512)

Table S4: Transformation functions layer by layer of the R2-Attention UNet architecture
